# Some Remarks on the Supposed Effects of Lime and Magnesia in Promoting the Solubility of Peruvian Bark

**Published:** 1786

**Authors:** Ralph Irving


### VIII. *Some Remarks on the supposed Effects of Lime and Magnesia in promoting the Solubility of Peruvian Bark. Communicated in a Letter to Dr.* Simmons *by* Ralph Irving, *M. D.*

AS you have been pleafed, on the authority of Dr. Skeete, to introduce into the London Medical Journal * fome account of the effects of magnefia in promoting the folubility of the Peruvian bark, I flatter myfelf, Sir, from your known candour, and defire to promote every kind of ufeful medical inquiry, that you will readily permit me, through the channel of the fame valuable work, to lay before the public a few remarks on fome of Dr. Skeete's deductions, which, to me, feem to be not well founded.—As the experiments with magnefia, mentioned by that gentleman in his late publication, are intimately connected with thofe on the effect of lime water, as a menftruum of the bark, related in the fame work, I fhall beg leave to begin with fome obfervations on the latter.

From the red colour of an infufion of bark in lime water, from the remarkable bitternefs

* See page 254.

of its tafte, and from an equal meafure of it fcarcely weighing one grain more than an infufion in water, Dr. Skeete finds* a difficulty in explaining why the lime water had not diffolved a greater quantity of bark, in correfpondence to its fenfible properties, fo remarkably fuperior to thofe of an infufion in plain water. In attempting to folve this difficulty, I would premife that Dr. Skeete has neglected to take into the account the weight of the lime. This is furely an overfight, which muft diminifh the validity of his main pofition. With refpect to the colour, I can at pleafure produce it by adding a portion of lime water to a fimple infufion; but the addition of the lime water, inftead of rendering the folution more complete, does, in fact, precipitate a quantity of the fufpended matter. Now I apprehend no perfon will believe that a fubftance capable of precipitating the matter of an infufion, does add either to the ftrength or quantity of that matter. I would, therefore, account for the *red colour* of an infufion of bark in lime water, from the want of that matter, the depofition of which,

* Exp. and Obf. on Quilled and Red Peruvian Bark, page 38.

from from the ſimple infuſion, produces the red colour. This matter ſeems to be of a gummy nature, and capable of affecting the rays of light in ſuch a manner as to produce the paleneſs of the ſimple infuſion. I will alſo alledge that this matter is poſſeſſed of a ſheathing property; wherefore its abſence in the lime-water infuſion, joined to the diſagreeable taſte of the lime itſelf, are, in united force, ſufficient to account for the more intenſe taſte of Dr. Skeete's infuſion. But the Doctor, ſeeming to conſider the circumſtance of weight as the only obſtacle in eſtabliſhing the pre-eminence of the lime-water infuſion, has attempted to reconcile the whole by the following opinion: — "But if "the abſolute weight of the bark be not much "diminiſhed by the action of ſuch a men-"ſtruum, it certainly appears, from every "other trial, to have diſpoſſeſſed it of its "active properties in a proportion vaſtly ſu-"perior to common water *." But whence is this opinion of Dr. Skeete's? It is immediately deduced from the following experiment: — "A tea-ſpoonful, for inſtance," ſays he, "of

* Exp. and Obſ. on Quilled and Red Peruvian Bark, page 38.

"the solution of sal. martis being added to "some of the above infusion, immediately "rendered it very turbid, and dark coloured, "and soon occasioned a copious precipitation "of a blackish colour *." Now let any man, tolerably conversant with chemistry, pause but for a moment, and consider what takes place in the above experiment; will he not perceive at once that a single elective attraction, and a double precipitation, must be the certain consequence of such a mixture? Vitriolic acid having a much greater attraction for lime than for iron, the latter must, therefore, beyond all doubt, be precipitated. But farther, a gypsum, insoluble in that quantity of water, must result from the union of the lime and acid, and which, in its turn, must also be deposited. It is not, therefore, surprising that Dr. Skeete's liquor was turbid indeed; nor ought we to account for the colour of the precipitate from the action of bark alone, as by lime water itself we can obtain a ferrugineous precipitate equally black as that described by Dr. Skeete.

With respect to the effects of magnesia in increasing the solubility of the bark, the experi-

* Exp. and Obs. on Quilled and Red Peruvian Bark, page 39.

ments ments related by Dr. Skeete are, if I miſtake not, liable to the ſame objections as thoſe with lime water. The Doctor has himſelf proved a ſolution of part of the magneſia in theſe infuſions; but the vitriolic acid having a much greater attraction for magneſia than for iron, every one muſt perceive, at firſt glance, that the latter muſt therefore be precipitated, and that the clear liquor will be a ſolution of Epſom ſalt. We are, therefore, I think, as with lime in the lime-water infuſion, to account for the formation of ink from the preſence of the magneſia, much more than from the action of the bark.

The decompoſition which muſt take place by means of the acid in the ſtomach, and ſome other conſiderations, might afford various objections to the uſe of ſuch a mixture as bark and magneſia in the practice of phyſic; but as its alledged ſuperiority is chiefly deduced from the teſt of chalybeates, and I flatter myſelf I have ſhewn this to be not well founded, I am unwilling to trouble you at preſent with any farther reflections on the ſubject.

